# Supplementary material for: Tailoring and optimizing fatty acid production by oleaginous yeasts through the systematic exploration of their physiological fitness
Source: Microb Cell Fact. 2022 Nov 3;21:228. doi: 10.1186/s12934-022-01956-5 (PMC9632096; doi:10.1186/s12934-022-01956-5)
Supplement: Supplementary file 1 — Additional file 1. Calibration curves to calculate dry cell weight of C. oleaginosus and Y. lipolytica from OD600. [file 12934_2022_1956_MOESM1_ESM.docx]

**Supplementary Material**


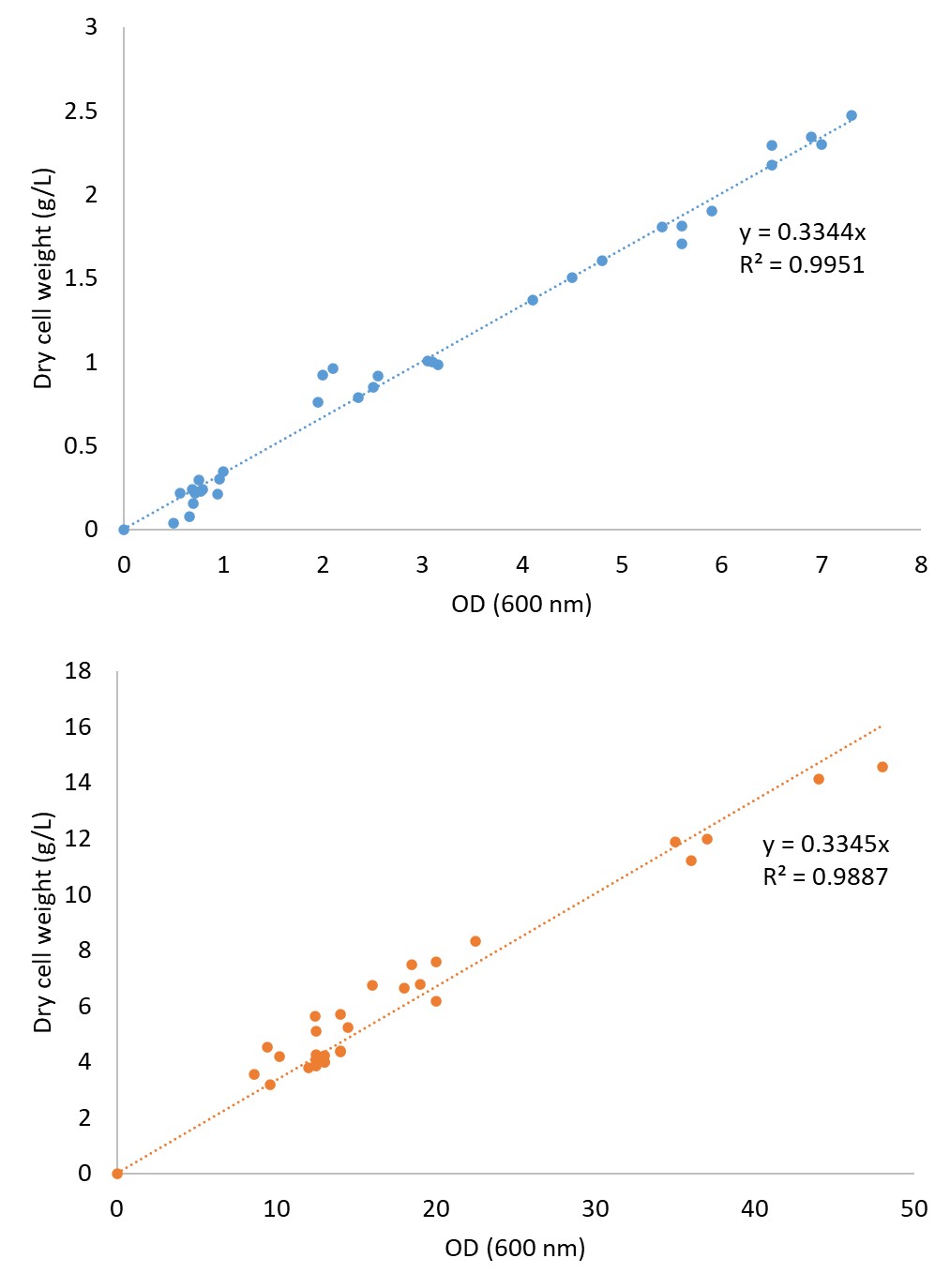
**a)**

**b)**

**Figure S1.** OD versus dry cell weight curve for a) *C. oleaginosus* and b) *Y. lipolytica*.
